# Small non-coding RNAs add complexity to the RNA pathogenic mechanisms in trinucleotide repeat expansion diseases

**DOI:** 10.3389/fnmol.2013.00045

**Published:** 2013-12-03

**Authors:** Eulàlia Martí, Xavier Estivill

**Affiliations:** ^1^Genomics and Disease, Bioinformatics and Genomics Programme, Centre for Genomic RegulationBarcelona, Spain; ^2^Universitat Pompeu FabraBarcelona, Spain

**Keywords:** small non-coding RNAs, trinucleotide repeat expansion, RNA-toxicity, miRNA, antisense small RNA

## Abstract

Trinucleotide-repeat expansion diseases (TREDs) are a group of inherited human genetic disorders normally involving late-onset neurological/neurodegenerative affectation. Trinucleotide-repeat expansions occur in coding and non-coding regions of unique genes that typically result in protein and RNA toxic gain of function, respectively. In polyglutamine (polyQ) disorders caused by an expanded CAG repeat in the coding region of specific genes, neuronal dysfunction has been traditionally linked to the long polyQ stretch. However, a number of evidences suggest a detrimental role of the expanded/mutant mRNA, which may contribute to cell function impairment. In this review we describe the mechanisms of RNA-induced toxicity in TREDs with special focus in small-non-coding RNA pathogenic mechanisms and we summarize and comment on translational approaches targeting the expanded trinucleotide-repeat for disease modifying therapies.

## INTRODUCTION

In the human genome trinucleotide repeats (TNR) are especially abundant in intergenic regions, gene introns and untranslated regions, and translated segments of protein coding genes. Short tandem-TNR have generally less than 30 copies in the normal population (Ellegren, 2004). Abnormal expansions of certain types of TNR result in trinucleotide repeat expansion diseases (TREDs), a group of inherited human genetic disorders involving the nervous system (**Table 1**; Orr and Zoghbi, 2007). Expansions of triplet repeats occur in coding or non-coding regions of unrelated genes and typically result in late-onset neurological diseases. Disease severity and onset are largely dependent on the expansion length. The pathogenic mechanisms associated to TNR expansions have been an extensive field of research over the last two decades. Different studies have revealed a tremendous complexity in the pathomechanisms, with diverse detrimental effects probably coexisting in cells. This complexity lies beneath the selective affectation of specific cell types in the brain, which is characteristic in each TRED.

**Table 1:** Trinucleotide expansion diseases.

Disease	Repeat type (normal vs expanded)	Gene	Gene function	Repeat in coding regions of the sense transcript	Repeat in non-coding regions of the sense transcript	Bidirectional-transcription	RAN translation	Small repeated CNG biogenesis	Described pathogenic process
Dentatorubral-pallidoluysian atrophy (DRPLA)	CAG (3–36/49–88)	*ATN1*	Nuclear receptor correpressor	PolyQ	-	Yes^*^	Unknown	Unknown	PolyQ gain of function
Fragile X tremor/ataxia associated syndrome (FXTAS)	CGG (6–52/60–200)	*FMR1*	Translation repressor, mRNA trafficking from the nucleus to the cytoplasm	-	5′UTR	Yes (CCG expansion in the *FMR1_AS*)	Yes (PolyG)	Yes	RNA foci sequestering MBNL, hnRNP G, hnRNPA2/B1, SAM68, Pur α , lamin A/C. Chromatin changes. Altered miRNA biogenesis
Friedeich ataxia (FRDA)	GAA (6–32/>200)	*FXN*	Biosynthesis of heme and assembly and repair of iron-sulfur clusters	-	Intron	Yes *FAST-1 *	Unknown	Unknown	FXN loss of function, chromatin changes
Huntington disease (HD)	CAG (6-35/36-121)	*HTT*	Transcription, intracellular signalling, trafficking, endocytosis, metabolism	PolyQ	-	Yes (CUG expansion in the *HTT_AS*)	Unknown	Yes	PolyQ gain of function, RNA foci sequestering MBNL, sCAG biogenesis and activity
Huntington’s disease like 2 (HDL2)	CTG (6–28/40–59)	*JPH3*	Formation of junctional membrane complexes, which link the plasma membrane with the endoplasmic or sarcoplasmic reticulum in excitable cells	PolyL; PolyA	3′UTR	Yes (CAG expansion in the *JPH3_AS*)	Unknown	Unknown	RNA foci sequestering MBNL
Myotonic dystrophy type 1 (DM1)	CTG (5–37/50 to >3500)	*DMPK*	Regulates the expression of muscle-specific genes	-	3′-UTR	Yes (CAG expansion in the DMPK_AS)	Yes (PolyQ)	Yes	RNA foci sequestering MBNL, CUGBP1 activation chromatin changes
Spinocerebellar ataxia 1 (SCA1)	CAG (6–39/39–81)	*ATXN1*	Gene expression regulation	PolyQ	-	Yes^*^	Unknown	Yes	PolyQ gain of function
Spinocerebellar ataxia 2 (SCA2)	CAG (13–33/>34)	*ATXN2*	Possible role in RNA metabolism	PolyQ	-	Yes^*^	Unknown	Unknown	PolyQ gain of function
Spinocerebellar ataxia 3 (SCA3)	CAG (13–44/>55)	*ATXN3*	Deubiquitination, transcriptional regulation	PolyQ	-	Yes^*^	Unknown	Unknown	PolyQ gain of function
Spinocerebellar ataxia 6 (SCA6)	CAG (13–44/>55)	*CACNA1A*	Calcium channel controlling neurotransmitter release and calcium homeostasis	PolyQ	-	Yes^*^	Unknown	Unknown	Protein GOF/LOF (?)
Spinocerebellar ataxia 7 (SCA7)	CAG (4–35/37–306)	*ATXN7*	Component of TFTC/STAGA transcriptional coactivator complexes, regulates retinal gene expression	PolyQ	-	Yes (CUG expansion in SCAANT1) (Sopher et al., 2011)	Unknown	Unknown	PolyQ gain of function
Spinocerebellar ataxia 8 (SCA8)	CTG (<50/74–1300)	*ATXN8*	Unknown	Non-coding RNA	Non-coding RNA	Yes (CUG expansion in the *ATX8_OS* 3′UTR)	Yes (PolyA, PolyQ)	Unknown	PolyQ gain of function ATX8; RNA gain of function ATX8_OS
Spinocerebellar ataxia 12 (SCA12)	CAG (4–32/51–78)	*PPP2R2B*	Negative control of cell growth and division	-	5′-UTR	Yes (CUG expansion in the*PPP2R2B_AS_*) (Brusco et al., 2002)	Unknown	Unknown	Unknown
Spinocerebellar ataxia 17 (SCA17)	CAG (25–42/47–63)	*TBP*	Initiation of transcritption	PolyQ	-	Yes^*^	Unknown	Unknown	PolyQ gain of function

* Detected according to ASSAGE sequencing in normal peripheral blood monocyteic cells (He et al., 2008). Deeper characterization of the antisense transcript is not available.

The largest group of inherited polyglutamine (polyQ) disorders is caused by expansions of CAG repeats in the open reading frame (ORF) of unique genes, including the Huntington’s disease (HD) genes and several spinocerebellar ataxias (SCA) genes. In these diseases the predominant hypothesis has been that the expanded polyQ track confers detrimental properties to the protein, that compromise cell homeostasis. The consequences of polyQ expansion in the HTT protein have been systematically characterized, with detrimental effects in transcriptional activity, vesicle trafficking, mitochondrial function and proteasome activity (Zheng and Diamond, 2012). However, the view of a protein-based toxocity in polyQ disorders has been challenged, as recent findings point to an additional toxic effect of the expanded CAG in the exon 1 of *HTT* mRNA (Banez-Coronel et al., 2012).

TNR expansions also occur in non-translated regions of selective genes. In myotonic dystrophy (DM1) a CTG expansion in the 3′UTR of the *DMPK* gene (50–3000 repeats) leads to neuromuscular degeneration (Brook et al., 1992). A CGG expansion (above 200 repeats) in the 5′-untranslated region (5′UTR) of the *FMR1* gene produces fragile X syndrome, the most common type of mental retardation. Yet, shorter CGG expansions (55–200 repeats) are associated to different pathologies such as fragile X tremor/ataxia syndrome (FXTAS) and primary ovarian failure (POF; Verkerk et al., 1991; Hagerman and Hagerman, 2004). Expansions occurring in non-translated regions produce RNAs with a toxic gain of function, involving a number of mechanisms, described in subsequent sections.

The recent discovery of repeat associated non-ATG (RAN) translation (Zu et al., 2011) has changed the view of TREDs pathogenesis, as toxic proteins may be also produced from expanded TNR thought to be embedded in non-coding RNAs. RAN-translation from Ataxin8 Oposite Strand (*Ataxin8_OS*) with an expanded CAG has been shown in different frames, in SCA8 mouse models and in patients with SCA8 (Zu et al., 2011). The same study showed RAN-translation across DM1 transcripts, resulting in the accumulation of PolyQ expanded proteins in DM1 mice models myoblasts and cardiomyocites. A similar phenomenon has been recently demonstrated in expanded CGG repeats in *FMR1* 5′-UTR (Todd et al., 2013). A cryptic polyglycine-containing protein (FMRpolyG) was detected accumulating in ubiquitin-positive inclusions in Drosophila, cell culture and mouse disease models, and in brains of patients with FXTAS. The relevance of this mechanism needs to be specifically addressed for each TRED.

In this review we focus on the RNA pathogenic mechanisms in TREDs. We present the existing evidences for RNA binding protein (RBP) sequestration by different expanded TNR and the linked altered biological processes. We address the possible relevance of bidirectional transcritption in TREDs loci and further discuss about the role of small non-coding RNAs in TREDs pathogenesis. Finally, we summarize the latest therapeutic strategies in TREDs, based on selective targeting of the allele with the expanded TNR.

## MECHANISMS OF RNA-TOXICITY IN TREDs

Recent findings indicate that alterations of RNA sequences can lead to abnormal RNA–protein interactions, alteration of protein translation, or RNA interference (RNAi) activation, among other anomalous processes. These altered pathways contribute to disruption of normal cell function and homeostasis, eventually leading to cell degeneration.

### TRINUCLEOTIDE REPEAT EXPANSIONS MODIFY ALTERNATIVE SPLICING EVENTS

**FIGURE 1:**
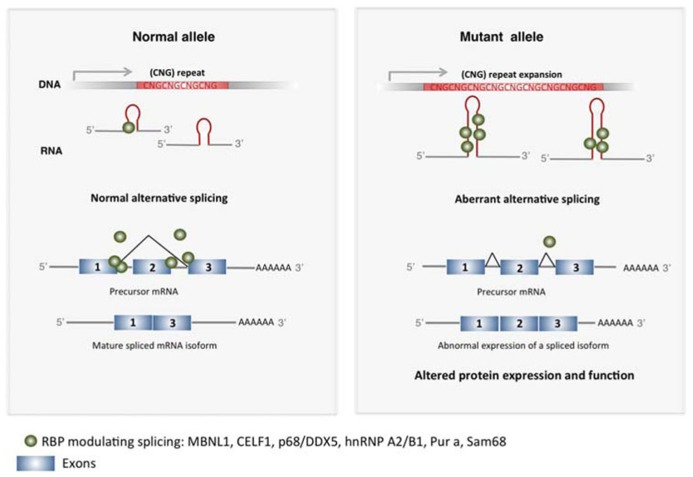
**Expanded TNR induce altered mRNA splicing in TREDs. ** Expanded CNG repeats sequester RBP in the nucleus that modulate exon usage during pre-mRNA maturation, including MBNL1, CELF1, p68/DDX5, hnRNP A2/B1, Pur a, Sam68. This results in altered alternative splicing in several genes, producing misslocalized or dysfunctional proteins that contribute to pathogenesis in TREDs.

In TREDs abnormal expanded TNR RNA–protein interactions results in disrupted protein conformation and inclusion formation. Sequestration of RBP by the expanded TNR leads to a loss of function of such proteins (**Figure 1**). Muscleblind-like splicing regulator 1 (MBNL1) and Elav-like family member 1 (CUGBP1 or CELF1) are regulators of mRNA splicing that present affinity for CUG and/or CAG repeats. In DM1, long CUG repeats lead to decreased MBNL1 activity and increased CELF1 activity in muscle cells, which results in mis-splicing events in different developmentally regulated genes including the insulin receptor (*IR*), the chloride channel (*CLCN1*) and the cardiac tropin T (*TNNT2*), which explain several aspects of DM1 symptomatology (Ranum and Cooper, 2006; Wheeler and Thornton, 2007).

Aberrant splicing of the bridging integrator-1 (*BIN1*) pre-mRNA has been recently described in DM1 (Fugier et al., 2011). BIN1 protein is required for the biogenesis of muscle T tubules, essential for excitation-contraction coupling. Mis-splicing of *BIN1* linked to a loss of function of MBNL1, produces an inactive form of BIN1 protein. While direct interaction of MBNL1 with the CUG repeat depletes MBNL1, increased levels of CEFL1 are the consequence of an indirect effect of CUG expansion involving PKC-pathways (Kuyumcu-Martinez et al., 2007). CELF1 hyper-phosphorylation mediated by PKC, leads to its increased stability and activity (Kuyumcu-Martinez et al., 2007). Splicing alterations have been also reported in DM1 and SCA8 brains, with neuronal cells showing MBNL1 nuclear inclusions (Jiang et al., 2004; Daughters et al., 2009; Mykowska et al., 2011). Abnormal splicing of multiple exons in microtubule associated protein tau (*MAPT*), and exon 7 in the amyloid precursor protein (*APP)* and exon 5 in glutamate receptor *NMDAR1*, have been detected in brains of DM1 patients and mouse models (Jiang et al., 2004; Gomes-Pereira et al., 2007) and analogous splicing alterations have been shown in SCA8 mice (Daughters et al., 2009) which may explain neurological alterations in these diseases. The relevance of expanded RNA-MBNL1 interaction in DM1 is strengthened in a mouse model expressing an expanded CUG RNA that recapitulates DM1 phenotypes. In this model, partial recovery of the mis-splicing defects is achieved by reestablishing MBNL1 levels. Another recently described repeated-CUG interactor is the p68/DDX5 helicase, which is present in mutant *DMPK* foci in DM1 (Laurent et al., 2012). p68/DDX5 modifies of MBNL1 splicing activity and has been proposed to influence pathogenicity in DM1.

The CGG expansions in the FMR1 5′UTR causing FXTAS may also sequester MBNL1, which is accumulated as abnormal inclusions in brain of FXTAS patients (Iwahashi et al., 2006). Other RBPs with specific affinity for CGG repeats are Pur-α and hnRNP A2/B1, which have been found in inclusions in FXTAS models (Jin et al., 2007; Sofola et al., 2007). In addition, CELF1 indirect binding to the CGG repeats through the RBP hnRNP A2/B1, leads to its loss of function (Sofola et al., 2007). Loss of function of Pur-α and hnRNP A2/B1 induce neurological alterations in mice (Khalili et al., 2003), suggesting their participation in FXTAS neuropathology. Recently, loss of function of the splicing factor Sam68 through binding to expanded CGG repeats has been shown in FXTAS patients, contributing to aberrant splicing of the ATPase *ATP11B* and the Survival of Motor Neuron 2, centomeric *SMN2* genes (Sellier et al., 2010).

Together, these data indicate that loss of function of RBP that have affinity for expanded TNR is a common mechanism operating in TREDS and further suggest that full characterization of the set of RBP binding to different types of TNR expansions will provide insights into specific pathogenic processes.

### TRINUCLEOTIDE-REPEAT EXPANSIONS ALTER miRNA BIOGENESIS

**FIGURE 2:**
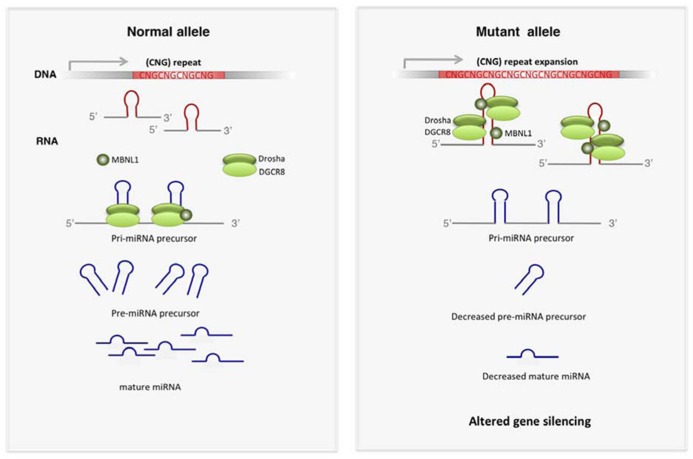
**Altered miRNA biogenesis in TREDs. ** Expanded CNG repeats have affinity for key players of miRNA biogenesis such as DROSHA and DGCR8. Other proteins modulating the activity of DROSHA complex, such as MBNL1, p72 and p68, are also sequestered by expanded TNR. Functional depletion of these proteins leads to decreased production of mature miRNAs, which results in increased expression of miRNA targets.

The most recent findings suggest that sequestration of RBP by TNR expansions has other consequences in addition to alternative splicing perturbations (**Figure 2**). MBNL1 and the RNA helicases p68 and p72 influence the activity of proteins involved in microRNA (miRNA) biogenesis (Fukuda et al., 2007; Rau et al., 2011). MBNL1 in normal conditions binds to pre-miR-1 precursor, allowing the normal production of mature miR-1. Depletion of MBNL1 in DM1 permits the activity of the processing regulator LIN28, which binds to pre-miR-1 and promotes 3′-end uridylation, thus resulting in inhibition of pre-miR-1 processing by the endonuclease Dicer. The disruption of the normal pre-miR-1 processing by MBNL1 loss of function results in increased levels of miR-1 targets, including the calcium channel CACNA1C and the gap-junction channel GJA1, which may contribute to the cardiac defects in DM1 (Rau et al., 2011). The modulatory role of p68 and p72 helicases in the miRNA-processing complex (Fukuda et al., 2007) suggest that analogous mechanisms may exist for these proteins.

Alterations in miRNA biogenesis pathways have been also described in FXTAS (Sellier et al., 2013). The hairpin structure of the expanded CGG in the FMR1 5′UTR mRNA mimics the structure of the miRNA-precursors (pri-miRNAs). DGCR8 and its partner DROSHA, key players in miRNA-precursor processing, are sequestered by CGG-RNA repeats (**Figure 2**). Depletion of these processors compromises the biogenesis of many miRNAs, thus triggering downstream detrimental gene expression perturbations, which likely contribute to FXTAS pathogenesis. However, this mechanism may be tissue-dependent, as small RNA profiling in peripheral blood of FXTAS patients does not reveal a general miRNA downregulation (Alvarez-Mora et al., 2013).

General perturbations in miRNA biogenesis result in altered mature miRNAs expression and subsequent modifications of gene silencing, which likely contribute to disrupted cell homeostasis in TREDs.

### BIOGENESIS AND ACTIVITY OF SMALL REPEATED CNG IN TREDs SENSE TRANSCRIPTS

**FIGURE 3:**
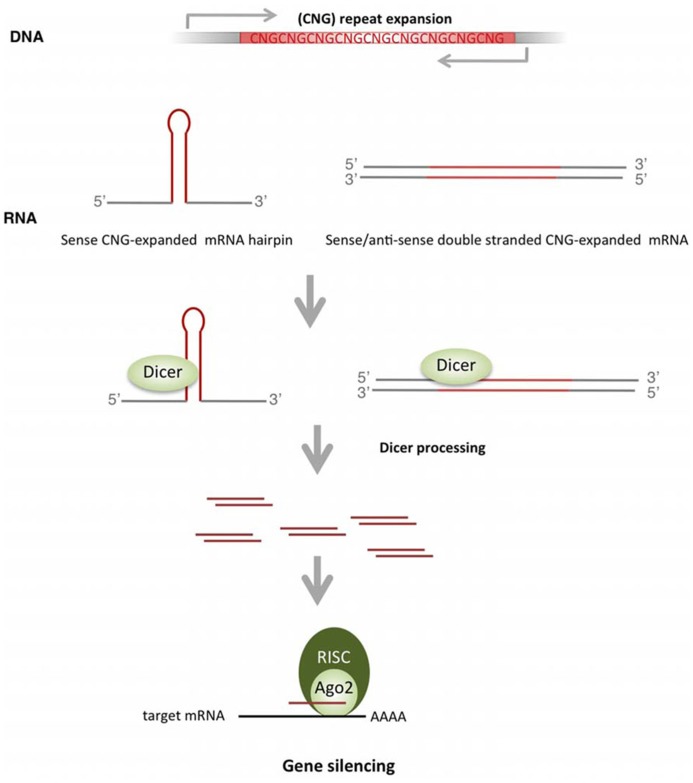
**Biogenesis and activity of sCNG in TREDs. ** TNR expansions produce hairpin-like structures that are recognized and cleaved by Dicer to form small RNAs with repeated TNR (sCNG). Bidirectional transcription though the expanded TNR offers another source of sCNG, where perfectly complementary double stranded mRNAs with expanded TNR are cleaved by Dicer to form sCNG. sCNG are incorporated into the RISC complex and silence genes with partial or perfect complementarity.

The hairpin structure of expanded CNG repeats (Galka-Marciniak et al., 2012) constitutes a substrate for Dicer, an endonuclease involved in miRNA biogenesis that excises RNA precursors to generate the mature short miRNA (**Figure 3**). Dicer recognizes the expanded triplet and cleaves it, producing small repeated RNAs (sCNG). *In vitro* approaches demonstrated that single stranded CGG-RNA constructs are cleaved by Dicer, producing short CGG-RNAs of approximately 21 nt (sCGG; Handa et al., 2003). Recombinant Dicer also cleaves long transcripts containing other types of long CNG repeats (CAG, CUG, CCG; Krol et al., 2007). Importantly, the Dicer-dependence of sCNG biogenesis has been demonstrated in fibroblasts of patients with DM1 (sCUG), HD (sCAG) and SCA1 (sCAG; Krol et al., 2007). In this study the authors further showed that sCNG were active as transcriptional inhibitors, since they downregulated the expression of transcripts with complementary target sequences. This inhibitory activity was dependent on Ago2, a key member of the RNA silencing machinery (Krol et al., 2007).

The relevance of sCNG in TREDs pathogenesis has been recently addressed in HD (Banez-Coronel et al., 2012). This study confirmed the biogenesis of sCAG in a neuronal cell model expressing expanded *HTT* exon-1, and in brain samples of patients with HD or the R6/2 HD mouse model. Importantly, the fraction of small RNAs (sRNAs) derived from cells expressing expanded *HTT* exon-1 produced neuronal death. Both the biogenesis and the toxic activity of sCAG were dependent on Dicer. Similarly, transfection of sRNAs isolated from the striatum and cortex of patients with HD induced significant neuronal toxicity. This toxic effect was prevented by oligonucleotides complementary to short CAG, strongly suggesting a detrimental effect of sCAG. Furthermore, toxicity may depend on downstream silencing effects, as HTT exon-1-derived sCAG were loaded onto Ago2 complexes and knocking-down of Ago2 prevented damage. However, the sequence/structure requirements for effective silencing of sCAG-targets remain to be resolved, since similar moderate inhibition was detected in luciferase assays performed with targets harboring a perfect sCAG-complementary CTG repeat or a CAG repeat that offers an interrupted sCAG target site. Whether the detrimental properties of sCAG include silencing effect through mRNA degradation and/or translational repression, or perturb gene expression networks through other mechanisms, remains to be determined. Interestingly, this study shows that the effect of sCAG-RNAs differed depending on the cell type, with high toxicity detected in BDNF-differentiated neuroblastoma cells. In this scenario, sCAG activity may provide a mechanism contributing to tissue selective affectation. The relevance of sCNG biogenesis and activity in other TREDs is an interesting field for future studies.

### BIDIRECTIONAL TRANSCRIPTION IN TREDs LOCI PRODUCE NEW PATHOGENIC PLAYERS

Much of the transcriptome is transcribed in both directions (Chen et al., 2004). While recent data suggest that only a small part of the sense transcript produces proteins (Derrien et al., 2012), the anti-sense transcripts, normally less abundant, are involved in the regulation of gene expression (He et al., 2008; Morris et al., 2008; Yu et al., 2008; Batra et al., 2010). Bidirectional transcription has been detected in many TREDs loci including, DM1, SCA8, FXTAS, SCA7, HDL2, and HD, suggesting a role in disease pathogenesis (**Table 1**; Cho et al., 2005; Ladd et al., 2007; Batra et al., 2010; Chung et al., 2011; Sopher et al., 2011; Wilburn et al., 2011; Seixas et al., 2012). Thus, TREDs pathogenic mechanisms typically associated with expanded toxic RNA may be complemented with those induced by abnormal expanded peptides that result from coding-antisense transcripts or by a complementary non-coding expanded RNA. For instance, in SCA8, the progressive cerebellar degeneration inducing ataxia is the consequence of a CUG expansion in the 3’ end of the non-protein coding *Ataxin 8OS* mRNA (Koob et al., 1999; Day et al., 2000). This led to the conclusion that the pathogenic mechanism was related with an expanded CUG-RNA toxic gain of function. Subsequently, bidirectional transcription was demonstrated in transgenic mice expressing the entire human locus with either normal or expanded CTG allele (Moseley et al., 2006). A progressive neuronal loss was found in the lines expressing expanded CUG, with concomitant co-expression of two transcripts in opposite directions. The sense transcript produced a non-coding CUG-expanded transcript (*Ataxin 8OS*) and an anti-sense transcript resulted in a CAG expansion that was translated into a highly enriched polyQ track (*Ataxin 8*). Intranuclear inclusions immunopositive for anti-polyQ antibodies, which are typical from PolyQ diseases and CUG foci formation co-localizing with MNBL1 were detected in cerebellar cells of the mouse model and patients with SCA8 (Daughters et al., 2009). Thus, both RNA and protein toxic gain of function may account for SCA8 pathogenesis.

A similar process could account for HDL2 pathogenesis that is caused by a CUG/CAG repeat expansion at the Juctophilin-3 (JPH3) locus (Holmes et al., 2001), The alternatively spliced forms in the *JPH3* gene place the CUG expansion in the polyleucine or polyalanine ORFs or in the 3′UTR. A *JPH3* transcript with expanded CUG repeats produce RNA foci that co-localize with MNBL1 and induces cell toxicity (Rudnicki et al., 2007). The existence of an anti-sense CAG transcript in the JPH3 locus was recently demonstrated, which may account for the detected polyQ proteianceous inclusions (Wilburn et al., 2011).

Anti-sense transcripts spanning the CGG repeat have been described in the FMR1 locus (*FMR1AS*) in human lymphoblastoma cells (Ladd et al., 2007). *FMR1AS* is spliced, processed and exported from the nucleus. The regulation of *FMR1AS* expression is dependent on CGG expansion size; being silenced in full CGG mutations (CGG > 200 nt), similar to the *FMR1* sense transcript. A recent study suggested that elevated expression levels of the sense and antisense expanded *FMR1* involving mitochondrial dysfunction participate in parkinsonism phenotype that is associated with CGG-repeat moderate expansions (Loesch et al., 2011). Thus, both *FMR1* and *ASFMR1* may contribute to the variable phenotypes associated with the CGG repeat expansion.

Anti-sense transcription at the DM1 locus has also been reported (Cho et al., 2005). Both sense and antisense transcripts extending across the CAG repeat were found in independent nuclear foci in a mouse model carrying >1,000 CTG repeats in the DM1 locus and in human tissues (Huguet et al., 2012).

HTT anti-sense (*HTTAS*) transcripts have been identified that contain the repeated CAG track (Chung et al., 2011). Repeat expansion reduces *HTTAS* promoter efficiency, and therefore *HTTAS* expression is reduced in the brain of HD patients. Through knocking down the *HTTAS* transcript the authors demonstrated its regulatory activity on HTT expression. The relevance of this regulatory mechanism in HD has not been addressed. However, a possibility exists that *HTTAS* provides an expanded CUG-based pathogenic mechanism.

One interesting mechanism derived from anti-sense transcription of genes containing TNR expansions is the activation of silencing mechanisms (**Figure 3**). Complementary repeats can form double-stranded structures compatible with endonuclease Dicer slicing activity. This results in the formation of short repeated RNAs that are incorporated into the RISC complex, possibly driving downstream gene silencing with detrimental consequences. This mechanism has been proven in a DM1 Drosophila model, in which the toxic effect of an expanded CUG track was largely enhanced if co-expressed with a CAG expansion. The co-expression of sense and anti-sense transcripts lead to the formation of repeat-derived small interfering RNAs in a process dependent of Dicer-2 and Ago-2 (Yu et al., 2011). Similarly, flies models expressing CAG/CUG -100 nt double stranded RNAs (Lawlor et al., 2011) showed a Dicer 2-dependent progressive neurodegenerative phenotype.

From these results it seems clear that small double stranded RNAs are detrimental for neuronal cells. However, the relevance of bidirectional transcription derived-siRNA in human disease needs to be proven, as the expression of antisense transcripts is normally low, which may limit the formation of these products.

In summary, bidirectional transcription through repeat regions of TREDs genes likely increases the complexity of the pathogenic mechanisms underlying the disease process, including the sequestration of different RBP and the biogenesis of small repeated TNR RNAs with silencing activity.

## EVIDENCES FOR RNA TOXICITY IN POLYQ DISORDERS

Although in polyQ diseases pathogenesis has been traditionally linked to altered function of the protein, a number of evidences suggest a complementary detrimental role of the expanded RNA.

*In vitro* structure determinations of expanded CAG repeats in the mRNA context of the *HTT, ATXN1-3, ATN1 *and AR genes that cause different polyQ diseases (**Table 1**) show compatibility with double stranded hairpin formation (Galka-Marciniak et al., 2012). Biochemical studies further suggested that MBNL1 has similar affinity for RNA containing either CUG or CAG repeats (Yuan et al., 2007). Although CAG expansions in polyQ diseases occur in the protein coding sequence, nuclear RNA inclusions accumulating MBNL1 have been detected in fibroblasts of patients with ATXN3 and HD (de Mezer et al., 2011; Mykowska et al., 2011). Alternative splicing defects similar to those observed in DM1 have been shown in these cells, suggesting that splicing alterations are likely the consequence of MBNL1 sequestration.

Expanded CAG repeats were shown to induce *in vivo* toxicity at the RNA level in Drosophila, *C. elegans* and mouse models. The *in vivo* evidence for repeated CAG RNA toxicity was first obtained in a Drosophila model of SCA3 (Li et al., 2008). The expression of untranslated CAG repeats of pathogenic length led to neurodegeneration in the absence of a mutant polyQ protein. The expression of translated CAA or interrupted CAG repeats resulted in a less severe phenotype than the expression of translated pure CAG repeats, which supported the importance of RNA structure for toxicity.

The CAG repeat toxicity at the RNA level was also demonstrated in a worm system (Wang et al., 2011). Both CAG and CUG repeats of pathological length were shown to form nuclear foci, in which the mutant transcript colocalized with the nematode ortholog of MBNL1, CeMBL. The disease phenotype was partially reversed by CeMBL over-expression.

The expression of untranslated long CAG repeats (200 copies) was also shown to be deleterious in transgenic mice (Hsu et al., 2011). Mice expressing EGFP transcripts with long CAG repeats in the 3′-UTR developed electrophysiological, histological and behavioral aberrations in the muscle. Detection of nuclear RNA foci in muscle cells in this model (Hsu et al., 2011) and in the striatum of the YAC128 HD mouse model expressing full-length human *HTT* (Pouladi et al., 2012) further suggests toxicity through expanded CAG-RNA.

These data indicate that cell failure in polyQ diseases may be the result of both an abnormal function of the protein harboring the expanded glutamine and the altered properties of the expanded-CAG RNA. The secondary structure of the CAG-repeat in each gene context and the dynamic expression and activity of RBP may provide specific pathogenic scenarios for cell dysfunction.

## miRNAs PERTURBTIONS IN TREDs

MicroRNAs are small RNA molecules of 20–24 nucleotides that generally inhibit the expression of target mRNA, by a mechanism involving mRNA degradation, translational inhibition or a combination of the two (reviewed in Esteller, 2011). miRNAs biogenesis involves processing of a primary transcript in the nucleus (pri-miRNAs) by the Drosha/DGCR8 microprocessor. This generates a precursor miRNA (pre-miRNA) that is exported to the cytoplasm by exoprtin-5, where the endonuclease Dicer cleaves it to release the double-stranded miRNA. One of these strands preferentially loads onto an RNA induced silencing complex (RISC), while the other strand is usually degraded. In animals, miRNAs recognize their targets through complementarity with the seed sequence (nucleotides 2–8 of the 5′ end of the miRNA). Hundreds of mRNA targets could exist per miRNA family and at least 30% of the mRNAs are targeted by miRNAs (O’Carroll and Schaefer, 2013).

miRNAs are fine-tuners of gene expression with key roles in the central nervous system function and development. The first evidences for a major role of miRNAs in neurons involved *in vitro* and *in vivo* models of loss of function of Dicer, a key limiting endonuclease in miRNA biogenesis. Dicer depletion disrupts the development of the CNS, with clear effects on brain morphology and cell-type specification and differentiation. Conditional knocking-down of Dicer in specific neuronal populations in adult mice further suggests a role of miRNAs in postmitotic long-term neuronal maintenance (Kim et al., 2007; Schaefer et al., 2007; Haramati et al., 2010). Furthermore, conditional loss of Dicer in astrocytes and oligodendrocyes causes neuronal dysfunction and degeneration (Shin et al., 2009; Tao et al., 2011). DGCR8 is one of the genes whose heterozygous deletion results in DiGeorge syndrome (Shiohama et al., 2003) with the majority of patients showing heart defects and developmental problems. The description of DGCR8 as key component of the microprocessor (Gregory et al., 2004) highlighted that defects in miRNA biogenesis likely underlie developmental defects. Haploinsufficiency of the microprocessor member DGCR8 also compromises neuronal viability in mice (Stark et al., 2008).

Perturbations of miRNA pathways have emerged as effectors of CNS damage, contributing to impaired cell homeostasis and neuronal death. MiRNAs deregulation produces alterations in the transcriptome that impact brain function, with consequences in neurodegeneration-relevant pathways, including inflammation, oxidative stress and mitochondrial integrity. The causes of miRNA expression deregulation are diverse, including changes in the activity of transcription factors or disease-associated genes and/or alterations in miRNA biogenesis or stability (Packer et al., 2008; Martí et al., 2010). Ischemia, excitotoxicity, oxidative stress or aging are examples of harmful stimuli producing alterations in the coding and non-coding transcriptome (Persengiev et al., 2011; Xu et al., 2012).

Several studies point to an involvement of miRNAs in the pathogenicity associated to TREDs. The toxicity of Ataxin-3 is enhanced upon Dicer ablation in Drosophila and human cell models. The administration of a pool of sRNAs restored Ataxin-3 toxic effect, suggesting a protective role of miRNAs (Bilen et al., 2006). Supporting a protective role of miRNAs in polyQ diseases, miR-34b was shown to mitigate the toxicity of Ataxin-3 in a Drosophila model (Liu et al., 2012).

The activity of disease-associated genes has emerged as one of the causes for miRNA deregulation in TREDs. Mutant HTT protein interacts with Ago2 in the P-bodies, and HTT depletion impairs miRNA mediated gene silencing (Savas et al., 2008). Strong miRNA deregulation has been detected in HD that may be in part associated with altered activity of the RE1-silencing transcription factor (REST). The expanded HTT polyQ track impedes sequestration of REST by wild-type HTT in the cytoplasm, therefore allowing its translocation to the nucleus. Mislocalization in the nucleus permits REST binding to RE1 repressor sequences thus decreasing neuronal gene expression, which triggers neuronal dysfunction. Several miRNAs with RE1 upstream binding sites are down-regulated in HD, including miR-9/miR-9* (Packer et al., 2008). A negative feed-back loop was proposed to occur in HD, involving the activity of REST-silencing complex that is regulated through the effect of miR9 and miR-9* on REST and Co-REST, respectively (Packer et al., 2008). High-throughput sequencing analysis has revealed strong miRNA expression deregulation in the striatum and frontal cortex of patients with HD (Martí et al., 2010). A significant enrichment of down-regulated miRNAs harboring upstream RE1 or P53 binding sites was also reported in this study, suggesting a major role of these transcriptional modulators in miRNA deregulation.

In another example, Ataxin-2 has been recently identified as a component of the miRNA pathway to regulate synapse-specific long-term-plasticity. This targets the putative relevance of ataxin-2/miRNAs in spinocerebellar ataxia neurodegeneration (McCann et al., 2011).

Significant miRNA deregulation has been also detected in pre-symptomatic versus symptomatic SCA1 mouse model cerebellum, which suggests a role of miRNAs in the evolution of the disease (Rodriguez-Lebron et al., 2013). In addition, the miRNA transcriptome has been also characterized in the muscle of a Drosophila model of DM1, expressing CTG repeats alone (Fernandez-Costa et al., 2013). Among the downregulated miRNAs, miR-1, miR-7, and miR-10 were confirmed in muscle of patients with DM1. Interestingly, over-expression of miR-10 extended the lifespan of CUG-expressing flies, suggesting a role in the disease.

Deregulation of miRNA specifically targeting dosage-sensitive disease genes may highlight their relevance as pathogenic biomarkers, which could be selectively targeted in therapeutic strategies. Dentatorubral-pallidoluysian atrophy (DRPLA) is caused by a CAG/polyQ expansion in DRPLA gene/protein, respectively. miR-200b and miR-429 target *REPRE* mRNA, whose protein product binds to DRPLA protein. Overexpression of REPRE induces DRPLA mislocalization. Thus, expression levels of miR-200b and miR-429 could potentially contribute to DRPLA (Yanagisawa et al., 2000; Karres et al., 2007). In another example, miR-886-3p targets the frataxin gene (*FXN*) that carries an intronic GAA. TTC triplet repeat expansion in Friedeich ataxia (FRDA; Mahishi et al., 2012). *FXN* mRNA and protein are decreased in FRDA. The authors found increased levels of miR-886-3p in blood and cells of patients with FRDA and further demonstrated that inhibition of miR-886-3p resulted in increases of *FXN* mRNA and protein. In addition, miR-19, miR-101 and miR-130 regulate Ataxin 1 (*ATXN1*) that causes spinocerebellar ataxia 1 (SCA1), when presenting a CAG expansion (Lee et al., 2008). The authors showed that inhibition of the activity of these miRNAs enhanced the cytotoxic activity of ATXN1 with an expanded polyQ in human cells, suggesting a miRNA mechanism modulating pathogenesis. More recently, it has been shown that, miR-144 and miR-101 play a central role in modulating the levels of *ATXN1* (Persengiev et al., 2011). In SCA1 patients and aging the levels of these miRNAs are increased, suggesting a role in neurodegeneration. Finally, the 3′UTR of the *FMR1* mRNA is targeted by miR-101, miR-129-5p, and miR-221 (Zongaro et al., 2013). Downregulation of miRNAs has been generally detected in the brain of patients with FXTAs (Sellier et al., 2013) and miR-221 is also downregulated in peripheral blood of males with FXTAS (Alvarez-Mora et al., 2013). Thus, deregulation of certain miRNAs may contribute to upregulation of expanded *FMR1*, which has been shown to participate in FXTAS pathogenesis.

These data indicate that altered expression of specific miRNAs may contribute to TREDs pathogenesis, directly perturbing the expression of dosage-sensitive genes that are essential in the maintenance of cell homeostasis. The dynamics of miRNA alterations may define the relevance of miRNA-pathways in disease evolution.

## THERAPEUTIC APPROACHES TARGETTING RNA-TOXICICITY

Several therapeutic targets in polyQ diseases such as HD involve the intervention of pathways perturbed by mutant polyQ proteins, including histone acetylation, excitotoxicity and oxidative stress (Clabough, 2013). Mutant HTT inhibits acetyltransferases, resulting in reduced levels of acetylated histones (Gray, 2010) Inhibition of histone deacetylase activity (HDACs) has been proposed as a therapeutic approach, alleviating altered gene expression produced by diminished acetyltransferase activity (Steffan et al., 2001; Gray, 2010). In addition, mitochondrial impairment and excitotoxicity have been involved in neuronal death in HD. Administration of antioxidants such as Coenzyme Q10 slow striatal atrophy in mouse models of HD (Beal, 2002) and delivery of growth factors and cytokines modify neuronal degeneration and prevents excitotoxic deficits in murine HD models (Mittoux et al., 2000; Perez-Navarro et al., 2000; de Almeida et al., 2001). Other strategies involve lowering the amount of mutant polyQ protein by reducing its production (see below) or enhancing its clearance. In this line, increased mutant HTT protein turnover in a mouse model improves disease outcome (Southwell et al., 2009).

**FIGURE 4:**
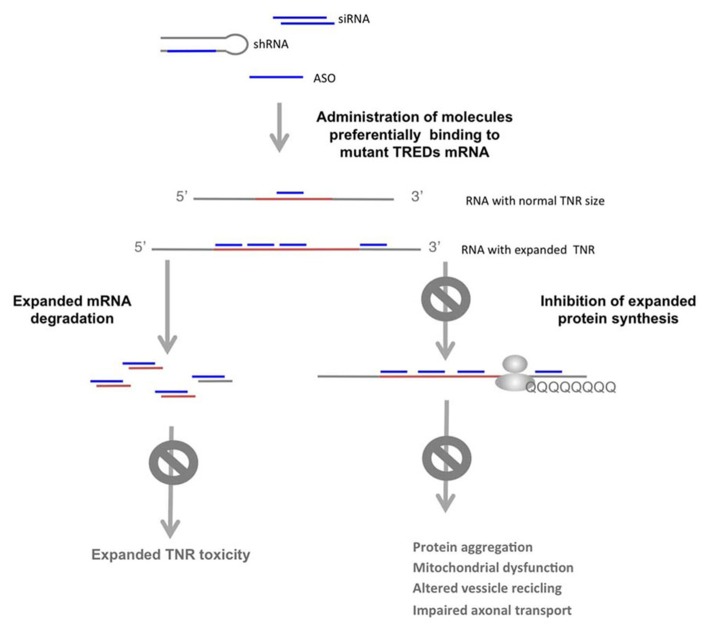
**Selective targeting of the expanded allele in TREDs. ** shRNA, modified ASO or siRNA directed against the expanded allele results in degradation of mutant mRNA or blockage of the mutant protein synthesis, which impedes the detrimental activities of the expanded TNR in the mRNA and the mutant protein. With this strategy the normal allele is not targeted, allowing its function.

Although targeting the mutant/expanded polyQ protein or its downstream pathogenic effectors improves disease readouts, these strategies may not impede expanded TNR RNA toxicity. Approaches aimed at blocking protein and/or RNA toxicity include the use of antisense oligonucleotides (ASOs) or RNAi (short hairpin RNAs, shRNA, double stranded siRNA or modified single stranded siRNAs), targeting the expression of the mutant gene (Sah and Aronin, 2011; Watts and Corey, 2012; **Figure 4**). RNAi using shRNA against mutant TREDs gene mRNA and protein have been successfully used *in vivo*. Intrastriatal adenoviral-delivery shRNA targeting mutant human HTT resulted in improved neuropathology and behavioral deficits in HD mouse models (Harper et al., 2005; Rodriguez-Lebron et al., 2005). Davidson’s lab later showed that miRNA expression systems to inhibit *HTT* were more efficient, overcoming unspecific toxic effects induced by shRNA expressing vectors (McBride et al., 2008; Boudreau et al., 2011). The therapeutic potential of 2′-O-(2-methoxy)ethyl modified ASOs targeting human *HTT* has been recently addressed (Kordasiewicz et al., 2012). The intracerebral transient infusion of these ASOs resulted in the RNAse H mediated degradation of the human *HTT* mRNA, in transgenic mouse models of HD (Kordasiewicz et al., 2012). Importantly, transient *HTT* reduction resulted in sustained motor and histopathological phenotypic reversal in the HD rodent models.

Because multiple studies suggest that reducing the expression of the wild-type allele may have deleterious consequences, selective targeting of the expanded allele should be optimal (Omi et al., 2005; Godin et al., 2010; Huang et al., 2011). In polyQ diseases, single-nucleotide polymorphisms (SNPs) linked to repeat expansions that distinguish wild-type from mutant alleles offer possibilities for specific targeting by RNAi. SNP targeting has been shown in HD (Schwarz et al., 2006; Carroll et al., 2011), SCA3 (Li et al., 2004) and SCA7 (Scholefield et al., 2009). Low frequency of allele-distinguishing SNPs in the human population limits this strategy. However, several authors have shown that targeting several specific SNPs can be applied to the majority of HD patients (Lombardi et al., 2009; Pfister et al., 2009; Warby et al., 2009).

Other potential therapeutic strategies in polyQ diseases, are based on the use of several types of modified single stranded ASOs that target the CAG expansion in the mutant *HTT* or *ATXN3* mRNA, while preserving the normal function of the wild type allele (Hu et al., 2009a,b,c; Gagnon et al., 2010; Fiszer et al., 2012; Yu et al., 2012). The longer CAG track in the mutant allele offers more binding sites for the complementary ASOs (**Figure 4**). In addition, the RNA structures of the expanded CAG that differ from those of the wild type allele (de Mezer et al., 2011; Krzyzosiak et al., 2012), may offer distinctive feature for preferential recognition of the mutant allele. In these studies, single-stranded ASOs containing locked nucleic acid (LNA) or peptide nucleic acid have been shown to selectively block the expression of mutant HTT at the protein level. This effect was not associated with extensive *HTT* mRNA degradation and LNA ASOs were shown to form stable structures with the target RNA. Although expanded RNA toxicity in HD has not been addressed, the formation of stable LNA ASOs : RNA duplexes has also the potential to block expanded CAG toxic effects in *HTT* mRNA.

Several studies have demonstrated a similar therapeutic potential of a CAG-repeat antisense or morpholino targeted to expanded-CUG in the *DMPK* mRNA (Mulders et al., 2009). In cell myoblast-myotube and patient cell models a 2′-O-methyl-phosphorothioate-modified (CAG)7 ASO silenced *DMPK* expression and reduced the ribonuclear aggregates. Intramuscular administration of these ASO in a DM1 mouse model further reduced expanded RNA toxicity (Mulders et al., 2009). In another study, a morpholino anti-sense (CAG)25 was shown to block the interaction of MBNL1 with the expansion in a mouse model, further dispersing nuclear RNA foci, preventing alterations in alternative splicing and preventing RNA toxicity. More recently, it has been shown that systemic administration of ASOs effectively knocked down the expression of nuclear retained-transcripts containing expanded CUG in the muscle, thus correcting the physiological, histopathological and transcriptional alterations associated to this DM1 model (Wheeler et al., 2012).

Together, these data suggest that in TREDs, targeting the expression and activity of the expanded allele both at the RNA and protein levels is a promising therapeutic strategy.

## CONCLUSION

RNA toxicity is a process underlying pathogenicity in TREDs, with TNR expansions occurring in both coding and non-coding regions of specific genes. Sequestration of transcriptionally active RBP, and RBP participating in miRNA biogenesis result in direct and indirect perturbations of the coding-transcriptome, which likely contribute to cell dysfunction. Full characterization of the repertoire of RBP in different types of TNR expansions is essential to understand common detrimental pathways in etiologically diverse neurological disorders. Studies that take into consideration the gene context may provide hints to understand disease specific aspects.

The biogenesis and activity of sCNG may contribute to TREDs pathogenesis. The gene silencing activity of these species likely trigger downstream detrimental effects, which may differ, depending on the cell type. This mechanism may complement the damaging activity of expanded protein and/or expanded RNA. Whether sCNG mechanisms are only related with gene silencing or present other activities and the real importance of sCNG mechanisms in each TRED remain to be determined.

In each TRED, the mechanistic bases for tissue specificity, with particular affectation of selective neuronal types, remain largely unknown. The different RNA-based pathogenic processes provide a number of scenarios that may underlie this specificity. These include the dynamic expression and cellular and subcellular localization of RBP, the amount of expression of the sense and anti-sense transcripts spanning the expanded TNR in each cell context, the regulation of the biogenesis and activity of sCNG in different brain areas and/or the temporal and spatial primary and secondary perturbations of the miRNA transcriptome.

The mechanistic complexity in TREDs stresses the need of additional studies to dissect the relative relevance of expanded protein- RNA-, and/or sRNAs-mechanisms in each disease. In this context, the use of modified ASOs or siRNA directed to the expanded TNR has the potential to block deleterious effects of expanded full-length RNA and derived sCNG, with concomitant inhibition of expanded protein expression.

## Conflict Of Interest Statement

The authors declare that the research was conducted in the absence of any commercial or financial relationships that could be construed as a potential conflict of interest.
